# Functional Analysis Identifies Multiple Effectors of *Candidatus* Liberibacter Asiaticus Suppressing Plant Pattern-Triggered Immunity

**DOI:** 10.3390/plants15020308

**Published:** 2026-01-20

**Authors:** Zhuoyuan He, Hongyan Li, Zonghui Zhao, Desen Wang, Hong Wu, Mei Bai, Xiangxiu Liang, Jian-Bin Yu

**Affiliations:** 1 College of Life Sciences, South China Agricultural University, Guangzhou 510642, China; hezy_scau@163.com (Z.H.); lihongyan@tdbio.com.cn (H.L.); hh020604@163.com (Z.Z.); wh@scau.edu.cn (H.W.); baimei924@scau.edu.cn (M.B.); 2 College of Plant Protection, South China Agricultural University, Guangzhou 510642, China; desen@scau.edu.cn

**Keywords:** effectors, virulence, plant immunity, *Candidatus* Liberibacter asiaticus, disease resistance

## Abstract

*Candidatus* Liberibacter spp. can infect most citrus plants and rely entirely on phloem sieve tube cells of the host plant for survival. *Candidatus* Liberibacter primarily contains *Ca.* L. asiaticus (CLas), *Ca.* L. africanus (CLaf), and *Ca.* L. americanus (CLam). Among these, CLas is the most harmful and widely distributed and is the primary pathogen of the devastating citrus disease Huanglongbing (HLB). Effectors are among the core weapons secreted by pathogens into plant cells to attack the plant immune system. In this study, we focused on CLas-specific effectors and those that are highly expressed during the infection stage to identify essential virulence effectors. Using secretion signal peptide prediction analysis, 40 candidate effectors with potential secretory capabilities were identified. Transient expression of these candidate effectors in *Nicotiana benthamiana* revealed their impact on pattern-triggered immunity, including INF-induced cell death and microbial pattern-induced reactive oxygen species (ROS) bursts, and the resistance of *N. benthamiana* to the bacterial pathogen *Pst* DC3000. 10 candidate effectors capable of suppressing plant immunity were identified. The stable expression of these candidate effectors in Arabidopsis showed that several candidate effectors enhanced plant susceptibility to *Pst* DC3000 and inhibited flg22-induced ROS production and MAPK activation. Among the three candidate effectors that significantly suppressed ROS burst, one effector, E3 (CLIBASIA_03085), interacts with the plant NADPH oxidase RbohD, a key enzyme responsible for ROS production. This suggests that E3 likely inhibits ROS accumulation by directly targeting RbohD. Here, we identified multiple candidate effectors capable of suppressing microbial pattern-triggered immunity that may be essential virulence factors for CLas infection, enhancing our understanding of CLas pathogenesis.

## 1. Introduction

*Candidatus* Liberibacter spp., belonging to the class Alphaproteobacteria, order Rhizobiales, and family Rhizobiaceae, can infect citrus plants and their relatives and other plants, including *Catharanthus roseus* and tobacco [1,2]. Its survival and reproduction are entirely dependent on the phloem sieve tube cells of host plants. The Liberibacter group includes three main species: *Candidatus* Liberibacter asiaticus (CLas), *Candidatus* Liberibacter africanus (CLaf), and *Candidatus* Liberibacter americanus (CLam) [3]. Among these, CLas is the most destructive and widely distributed, causing citrus Huanglongbing (HLB), a devastating disease that results in enormous losses to the global citrus industry [3,4]. CLas is a Gram-negative bacterium that is primarily transmitted by citrus psyllids. CLas has a wide geographical distribution and relatively high thermotolerance. This enables it to cause diseases in tropical, subtropical, and temperate citrus-growing regions such as Asia, North America, South America, and Africa [5]. CLas cannot be cultured in artificial media, and genetic manipulation remains extremely challenging. This has significantly hindered in-depth research on their physiological and biochemical characteristics and pathogenesis.

Plants rely primarily on their innate immune system to perceive and defend themselves against microbial pathogen invasion. Pattern recognition receptors (PRRs) on plant cell surfaces detect conserved pathogen-associated molecular patterns (PAMPs) to sense pathogen attacks, a process that often involves the participation of co-receptors [6]. The immune receptor FLS2, together with its co-receptor BAK1, recognizes bacterial flagellin (or its epitope flg22) [7]. Meanwhile, the receptor LYK4/5 forms a complex with its co-receptor CERK1 to perceive the fungal cell wall component chitin [8]. Upon pathogen perception, immune signals are transmitted to several key downstream regulatory components, including receptor-like cytoplasmic kinase (RLCK) family members (e.g., BIK1) [9,10], NADPH oxidases (e.g., RbohD) [11,12], and CrRLK1L receptor kinases (e.g., FERONIA) [13], thereby activating pattern-triggered immunity (PTI). Typical PTI responses include the reactive oxygen species (ROS) burst, activation of the MAPK signaling cascade, defense-related gene expression, and other immune responses [6]. Pathogenic microbes deliver effector proteins into host cells as virulence factors to target key components of immune signaling, suppressing plant immunity and facilitating infection [14]. Some effector proteins can be directly or indirectly recognized by intracellular nucleotide-binding leucine-rich repeat (NLR) receptors, leading to the activation of a stronger immune response known as effector-triggered immunity (ETI) [15]. Compared with ETI, PTI is generally more moderate and less easily evaded by pathogen evolution as a cornerstone of plant basal resistance.

Effector proteins are key virulence weapons for pathogens and play essential roles in promoting infection. For instance, the *Pseudomonas syringae* effector AvrPphB and *Xanthomonas* effector AvrAc proteolytically cleave and uridylate BIK1, respectively, thereby suppressing BIK1-mediated downstream immune signaling [10,16]. To exert their virulence functions, effectors must be delivered to host cells. For example, *Pseudomonas syringae* uses a type III secretion system to directly translocate effector proteins into the host cytoplasm [17]. CLas has both the Sec-dependent secretion system (Sec) and T1SS secretion pathways, with most of their effectors being secreted via the Sec pathway [3,18,19]. The Sec system recognizes the N-terminal signal peptides of effector proteins to facilitate their secretion into plant cells [4]. Several CLas effector proteins suppress plant immune responses. The CLas effector SDE1 targets citrus papain-like cysteine proteases (PLCPs), inhibiting PLCP-mediated disease resistance [20]. SDE3 interacts with citrus cytosolic glyceraldehyde-3-phosphate dehydrogenase (CsGAPC1/2), promoting the degradation of ATG8 family proteins and disrupting autophagosome formation, suppressing autophagy-mediated immunity [21]. Another CLas effector, SahA, exhibits salicylic acid (SA) hydroxylase activity and degrades SA and its derivatives, inhibiting SA-dependent defense signaling [22]. However, many key virulence effectors of CLas have yet to be identified. The characterization of these effectors is essential for a full understanding of CLas pathogenesis.

Identification of CLas effectors mainly relies on the prediction of secretory signal peptides. However, the limitations of early algorithms resulted in substantial discrepancies between different prediction tools and high false-positive rates. The recently developed SingalP 6.0 software uses a protein language model that enables accurate prediction of all five major signal peptide types [23]. Here, we focused on the previously reported CLas-specific effectors and those expressed during host infection. Using SignalP 6.0, we re-analyzed the secretory potential of these candidates and identified 40 candidate effectors. Through transient expression in *Nicotiana benthamiana* (*N. benthamiana*) and stable expression in transgenic Arabidopsis, we assessed their effect on plant PTI, identifying potential virulence effectors capable of suppressing host immunity, enhancing our understanding of CLas pathogenicity, and providing a theoretical basis for disease control strategies. This approach led to the identification of ten virulence effectors that significantly suppressed PTI responses. We found that the effector E3 (CLIBASIA_03085) likely targets the RbohD protein to inhibit the ROS burst, thereby attenuating host immunity.

## 2. Materials and Methods

### 2.1. Plant Materials and Growth Conditions

Arabidopsis and *N. benthamiana* plants were cultured at 22 °C and 65% relative humidity under a 10 h light/14 h dark cycle. The plants were cultivated in a growth medium composed of a 1:2 mixture of nutrient soil and vermiculite. Wild-type (Col-0) Arabidopsis plants used for *Agrobacterium tumefaciens*-mediated stable gene transformation were cultured at 24 °C and 60% relative humidity under a 14 h light/10 h dark cycle.

### 2.2. Prediction of Signal Peptide

The full-length amino acid sequences were used for signal peptide prediction. Signal peptides were predicted using SignalP 6.0 (https://services.healthtech.dtu.dk/services/SignalP-6.0/ (accessed on 1 September 2023) [23]. In the software settings, we selected “Other” for organism and chose “Long output” for output format.

### 2.3. Plasmid Construction

Total DNA was extracted from *Candidatus* Liberibacter asiaticus (CLas)-infected citrus leaves and used as a template for PCR experiments. The 40 predicted candidate effectors were amplified using specific primers and cloned into pCAMBIA1300-35S-HA-RBS vectors for transient expression in *N. benthamiana* or stable transformation in Arabidopsis. To perform the luciferase complementation image assays, the coding sequences of the indicated genes were cloned into the pCAMBIA1300-HA-Nluc-RBS or pCAMBIA1300-Cluc-RBS vectors. The strains and plasmids used in this study are listed in Table 1. The primers used are listed in Supplementary Table S1.

### 2.4. Cell Death Examination Assay

The cell death phenotype was examined using trypan blue staining and electrolyte leakage assays. To perform the trypan blue staining assays, leaves were boiled for 20–60 s in trypan blue solution (For 500 mL trypan blue solution: 0.4% trypan blue 20 mL, glycerol 50 mL, phenol 50 mL, lactic acid 50 mL, ethanol 314 mL, water 16 mL), incubated at 25 °C for 1–2 h, and de-stained in ethanol for 3 h. The cell death phenotype was visualized using photography.

To perform the electrolyte leakage assay, leaf disks were taken and floated with 5 mL sterile water at 25 °C for 3 h. The electrical conductivity was measured with a conductivity meter (METTLER TOLEDO, Zurich, Switzerland) and denoted as “value A”. Samples were boiled for 20 min and cooled to room temperature for electrical conductivity measurement, and denoted as “value B”. The final conductivity was calculated as (value A/value B) × 100% [30].

### 2.5. PAMP-Induced ROS Assay

Leaf disks were taken from Arabidopsis or *N. benthamiana* and incubated in sterile water overnight in a 96-well plate. The water was removed and samples were treated with approximately 200 μL luminescence detection buffer (20 μM luminol and 10 μg/mL horseradish peroxidase) containing 1 μM flg22 (SANGON, Shanghai, China) or 200 μg/mL chitin (C9752, Sigma, Burlington, MA, USA.), and luminescence was recorded using GLOMAX 96 microplate luminometer [24].

### 2.6. Pathogen Infection Assays

Arabidopsis at 4/5 weeks of age or *N. benthamiana* at 5/6 weeks of age were used for pathogen infection assays. The indicated *Pst* DC3000 strains were cultured in the KB medium, collected by centrifugation, resuspended in sterile water, and adjusted to a final concentration of 1 × 10^6^ CFU/mL. The bacteria were then infiltrated into the leaves of Arabidopsis or *N. benthamiana* using a needless syringe. Three days later, a leaf disk was collected using a 4 mm diameter punch and transferred to a 1.5 mL tube containing 100 μL ddH_2_O. After thorough grinding, an additional 900 μL ddH_2_O was added. The sample was then serially diluted, and 20 μL of the diluted suspension was spread onto TSA agar plates supplemented with rifampicin. Bacterial colonies were counted after incubation at 28 °C for 2 days [24].

### 2.7. Reverse Transcription-Quantitative Polymerase Chain Reaction (qRT-PCR)

Total RNA was extracted from plants using FastPure Plant Total RNA Isolation Kit (RC401-01, Vazyme, Nanjing, China). First-strand cDNA synthesis was performed using the HiScript III RT SuperMix for qPCR (+gDNA wiper) kit (R323-01, Vazyme), according to the manufacturer’s instructions. qRT-PCRs was performed using specific primers and the ChamQ SYBR qPCR Master Mix kit (Q311-02, Vazyme). The primers are listed in Supplementary Table S1.

### 2.8. Luciferase Complementation Image (LCI) Assay

Assays were performed as previously described [25]. Briefly, the indicated Nluc and Cluc constructs were introduced into *A. tumefaciens* strain GV3101, and the cultured bacteria were infiltrated into fully expanded *N. benthamiana* leaves for 2 days. Luciferin (1 mM, 12507, AAT Bioquest, Pleasanton, CA, USA) was injected into the leaves and a cooled CCD imaging apparatus was used to capture the LUC image.

### 2.9. Co-Immunoprecipitation Assay

The indicated constructs were transiently expressed in *N. benthamiana* by the *Agrobacterium*-mediated expression system for 2 days. Samples were collected, ground in liquid nitrogen and extracted using a protein immunoprecipitation (IP) buffer (50 mM HEPES [pH 7.5], 150 mM KCl, 1 mM EDTA, 0.3% Triton X-100, 1 mM DTT, proteinase inhibitor cocktail). After centrifugation, the supernatant containing the total plant protein extract was incubated with anti-FLAG M2 agarose for 2 h. Agarose were washed four times with protein IP buffer and eluted with 3 × FLAG peptide (Sigma, Burlington, MA, USA) for 45 min. The immunoprecipitates were separated by SDS-PAGE, and protein interactions were examined by Western blotting.

### 2.10. Bimolecular Fluorescence Complementation (BIFC) Assay

The indicated nYFP and cYFP constructs were transiently expressed in *N. benthamiana* by the *Agrobacterium*-mediated expression system for 2 days. The fluorescence was examined by a confocal microscope (ZEISS, Oberkochen, Germany).

## 3. Results

### 3.1. Prediction of Secreted Candidate Effectors in Candidatus Liberibacter Asiaticus

**Figure 1:**
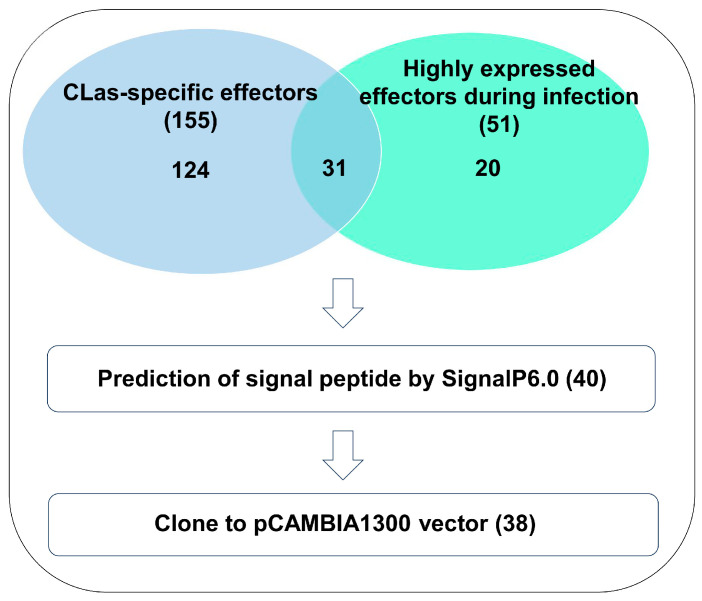
Prediction of candidate secreted effectors in *Candidatus* Liberibacter asiaticus (CLas) by SignalP 6.0.

Based on the published data, we conducted a statistical analysis of the number of effectors reported in the three *Candidatus* Liberibacter species. The results showed that 155 candidate effectors were unique to CLas, and 51 effector proteins were highly expressed during plant infection [18,31,32,33]. Among the CLas-specific effectors, 31 were highly expressed during infection, yielding 175 effectors that were either highly expressed or unique to the CLas genome (Figure 1).

Various tools, including SignalP 3.0, SignalP 4.1, and LipoP 1.0, have been used to predict the secretion-capable effectors. However, the limitations in their algorithms resulted in substantial discrepancies between the tools and high false-positive rates. The latest SignalP 6.0 uses protein language learning models capable of predicting all five types of signal peptide sequences with high accuracy and reliability [23]. In this study, we used the SignalP 6.0, an online tool to re-predict the signal peptides of these 175 effectors to assess their potential secretory capability (Figure 1 and Table S2). By applying a secretion probability threshold of >20%, 40 candidate effectors containing potential signal peptides were identified (Figure 1 and Table S3). These were designated as effectors 1–40 (E1–E40) and were considered candidate key virulence factors for further investigation (Table S3). The most highly expressed effectors were predicted to contain signal peptides (Table S2).

We successfully cloned 38 candidate effectors into HA-tagged pCAMBIA1300 vectors with E15 and E40 failures (Figure S1). Recombinant pCAMBIA1300 vectors were transformed into *Agrobacterium tumefaciens* strain GV3101, followed by transient expression in *N. benthamiana* leaves via infiltration. Protein immunoblot analysis after two days confirmed the successful expression of all candidate effectors except E11, which contained a frameshift mutation (Figure S1). Three candidate effectors (E12, E26, and E28) induced cell death in *N. benthamiana* (Figure S2A,B). In contrast, the expression of other candidate effectors did not cause any visible phenotypic changes (Figure S2C), making them suitable for subsequent studies on their effects on plant immune responses.

Analysis of published data revealed that 51 effectors from all three Candidatus Liberibacter species were highly expressed during host infection. Comparative analysis identified 155 CLas-specific effectors relative to *Candidatus* Liberibacter africanus (CLaf) and *Candidatus* Liberibacter americanus (CLam). Of these, 31 were both unique to CLas and were highly expressed during infection. SignalP 6.0 was used to re-predict signal peptides for these 175 effectors. Using a secretion probability threshold > 20%, we predicted 40 secreted effector proteins and named them candidate effector 1–40 (E1–E40). We successfully cloned 38 of these genes into the pCAMBIA1300 vector, except for excluding the *E15* and *E40*.

### 3.2. Impact of Candidate Effectors on INF1-Induced Cell Death

**Figure 2:**
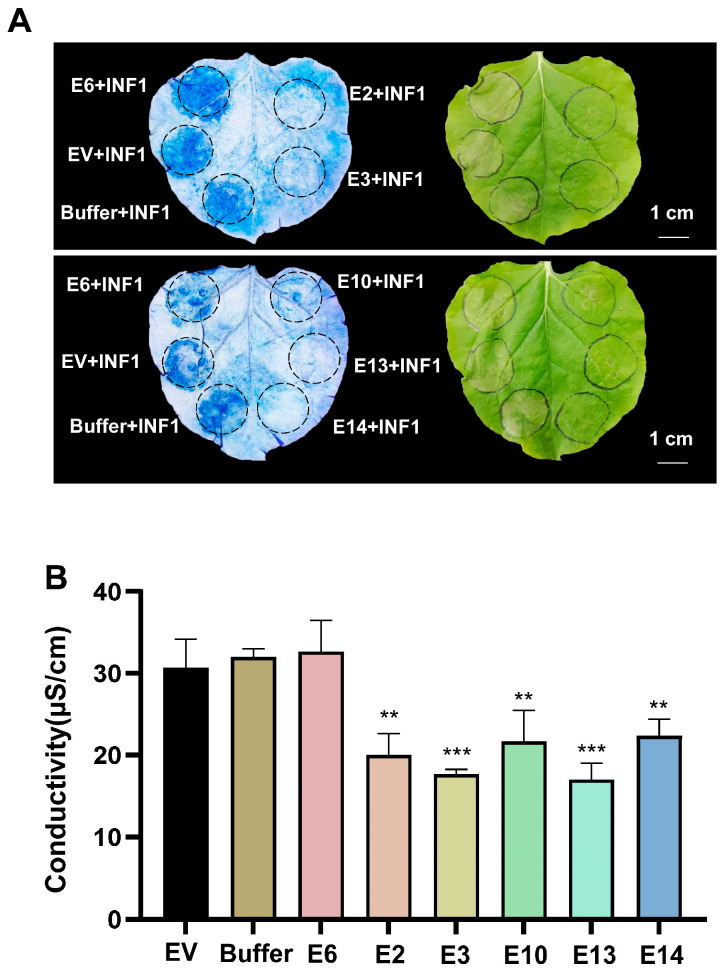
Analyses of the effect of transient expression of the candidate effectors on INF-induced cell death in *N. benthamiana.* (**A**,**B**) The indicated candidate effector was transiently expressed in *N. benthamiana* 1 day before infiltration of *Agrobacterium* containing the INF1 vector. The cell death phenotypes were visualized by trypan blue staining 3 days later (**A**). The related electrolyte leakage examination results were shown (**B**) (Mean ± SD, *n* ≥ 6, one-way ANOVA; ** *p* < 0.01, *** *p* < 0.001). The impact of all candidate effectors on INF1-induced cell death is shown in Supplementary Figure S3. Figure 2 presents the subset of five candidate effectors that significantly affected INF-induced cell death. E6 and EV (empty vector) were used as negative controls.

To evaluate the impact of candidate effectors on plant immunity, we transiently expressed all the candidate effectors in *N. benthamiana* leaves and assessed their effects on PTI immune responses. INF1, an oomycete-derived PAMP, is recognized by the PRR receptor REL and induces cell death in *N. benthamiana* [34,35]. The candidate effectors were transiently expressed in *N. benthamiana*. After 1 day, INF1 was infiltrated into the leaves, and the effect of effector pre-expression on INF1-induced cell death was assessed 3 days post-infiltration. Five candidate effectors (E2, E3, E10, E13, and E14) significantly suppressed INF1-triggered cell death (Figure 2A and Figure S3). Cell death is accompanied by ion leakage, leading to increased electrolyte conductivity. Conductivity measurements confirmed that these five candidate effectors also inhibited the INF1-induced increase in conductivity (Figure 2B), providing further evidence that they suppressed INF1-induced immune responses.

### 3.3. Impact of Candidate Effectors on PAMP-Induced ROS Burst

**Figure 3:**
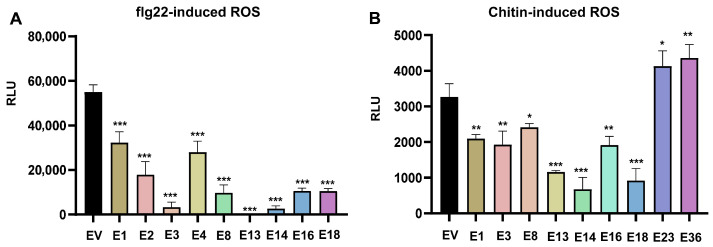
Analyses of the effect of transient expression of the candidate effectors on PAMP-induced ROS burst in *N. benthamiana.* (**A**,**B**) The indicated candidate effector was expressed in *N. benthamiana* plants by *Agrobacterium*-mediated transient expression for 2 days. ROS production induced by flg22 (1 μM) (**A**) and chitin (200 μg/mL) (**B**) was examined and peak relative luminescence unit (RLU) values are recorded (Mean ± SD, *n* ≥ 6, one-way ANOVA; * *p* < 0.05, ** *p* < 0.01, *** *p* < 0.001). The impact of all candidate effectors on flg22- and chitin-induced cell death is shown in Supplementary Figures S4 and S5. Figure 3 shows the effectors that significantly affected the PAMP-induced ROS burst.

PAMP treatment induces a rapid and robust burst of ROS, which is a hallmark of PTI activation [6]. Therefore, we investigated whether the candidate effector expression in *N. benthamiana* affects PAMP-triggered ROS production. We selected flg22, a bacterial-derived PAMP, and chitin, a fungal-derived PAMP, for these assays. Candidate effectors were transiently expressed in *N. benthamiana* leaves for 2 days, followed by measurement of the ROS burst induced by flg22 or chitin. Nine candidate effectors (E1, E2, E3, E4, E8, E13, E14, E16, and E18) inhibited the ROS burst triggered by the bacterial PAMP flg22 (Figure 3A and Figure S4). Meanwhile, seven (E1, E3, E8, E13, E14, E16, and E18) significantly suppressed fungal chitin-induced ROS accumulation (Figure 3B and Figure S5). Notably, E23 and E36 enhanced the chitin-induced ROS burst (Figure 3B and Figure S5). E3, E13, and E14 demonstrated broad-spectrum immunosuppressive activities by inhibiting all three PTI assays (Figure 2A,B and Figure 3A,B).

### 3.4. Impact of Candidate Effectors on Plant Disease Resistance

**Figure 4:**
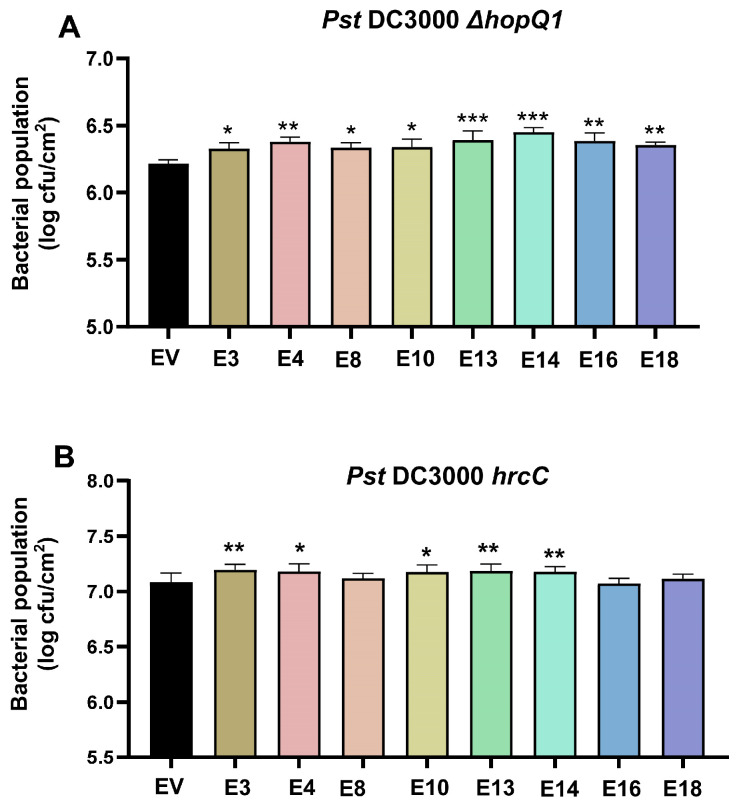
Analyses of the effect of transient expression of the candidate effectors on *N. benthamiana* disease resistance. (**A**) Analyses of the effect of transient expression of the candidate effectors on *N. benthamiana* resistance to *Pseudomonas syringae* pv. *tomato* DC3000 (*Pst* DC3000) *ΔhopQ1*. The indicated protein was expressed in *N. benthamiana* plants by *Agrobacterium*-mediated transient expression assay for 2 days. The leaves were infiltrated with *Pst* DC3000 *ΔhopQ1*, and the bacterial number was determined 3 days after inoculation. The impact of all candidate effectors on *N. benthamiana* resistance to *Pst* DC3000 *ΔhopQ1* is shown in Supplementary Figure S6. Figure 4A presents the subset of 8 effectors that significantly affected disease resistance (Mean ± SD, *n* ≥ 6, one-way ANOVA; * *p* < 0.05, ** *p* < 0.01, *** *p* < 0.001). (**B**) Analyses of the effect of transient expression of the eight candidate effectors on *N. benthamiana* resistance to *Pst* DC3000 *hrcC*. The indicated protein was transiently expressed in *N. benthamiana* leaves for 2 days, infiltrated with *Pst* DC3000 *hrcC,* and the bacterial number was determined 3 days after inoculation (Mean ± SD, *n* ≥ 6, one-way ANOVA; * *p* < 0.05, ** *p* < 0.01).

To further characterize the biological relevance of these observations, we examined disease resistance phenotypes using *Pseudomonas syringae* pv. *tomato* DC3000 (*Pst* DC3000) *ΔhopQ1*. HopQ1 is an effector capable of inducing a hypersensitive response in *N. benthamiana* [28]. Therefore, the *Pst* DC3000 *ΔhopQ1* mutant is used in bacterial infection assays. Candidate effector proteins are transiently expressed in *N. benthamiana* leaves. After 2 days, the plants were inoculated with the *Pst* DC3000 *ΔhopQ1* bacterial strain. Bacterial titers were measured 3 days post-inoculation. The study showed that eight candidate effectors (E3, E4, E8, E10, E13, E14, E16, and E18) compromise *N. benthamiana* resistance to *Pst* DC3000 *ΔhopQ1* to varying degrees (Figure 4A and Figure S6). We further induced the *hrcC* mutant strain of *Pst* DC3000, which lacks effector secretion capability, and provides a means to assess PTI [36]. E3, E4, E10, E13, and E14 significantly reduced the basal resistance against *Pst* DC3000 *hrcC* (Figure 4B), suggesting that these effectors may suppress plant PTI.

**Figure 5:**
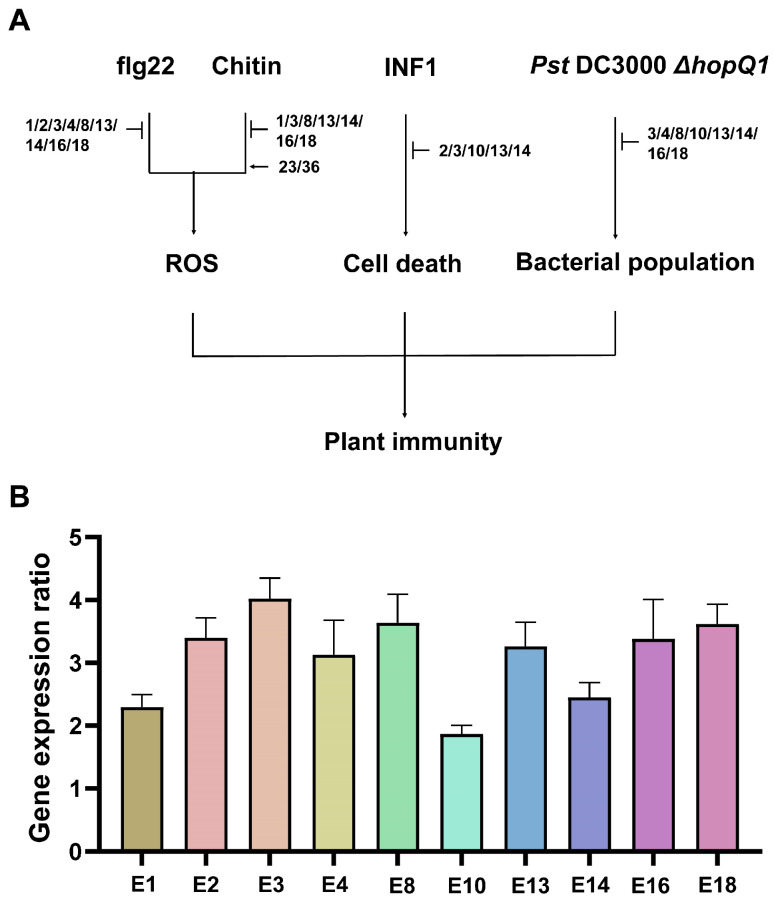
Summary of roles of candidate effectors in plant immunity. (**A**) Classification of candidate effectors based on their effects on immune responses and disease resistance to *Pst* DC3000 *ΔhopQ1*. Arrows indicate the promotion of immunity, and the T bars indicate the suppression of immunity. (**B**) Examination of the expression levels of 10 candidate virulence effectors in CLas-infected citrus leaves. Total RNA was extracted from CLas-infected and healthy citrus leaves and expression levels of the indicated effectors were examined by qPCR analysis. The ratios of expression levels between CLas-infected and healthy leaves were calculated and shown (Mean ± SD). The CLas *gyrA* gene (CLIBASIA_00325) was used as an internal control.

We then analyzed four experimental systems related to immunity and disease resistance, namely, INF1-induced cell death, flg22- and chitin-induced ROS burst, and resistance to *Pst* DC3000 *ΔhopQ1*. We identified ten candidate effectors (E1, E2, E3, E4, E8, E10, E13, E14, E16, and E18) that exhibited strong immunosuppressive activity in a specific assay or showed inhibitory effects across multiple assays (Figure 5A). Therefore, we designated these ten effectors as candidate virulence effectors (Table S2). All these candidate effectors were significantly upregulated during CLas infection in plants [18,31,32,33]. qPCR analysis confirmed that all candidate effectors were detectable in CLas-infected citrus leaves compared with healthy control leaves (Figure 5B), supporting their potential biological relevance during infection.

### 3.5. Immune Phenotypic Analysis of Transgenic Arabidopsis Plants Expressing Candidate Virulence Effectors

**Figure 6:**
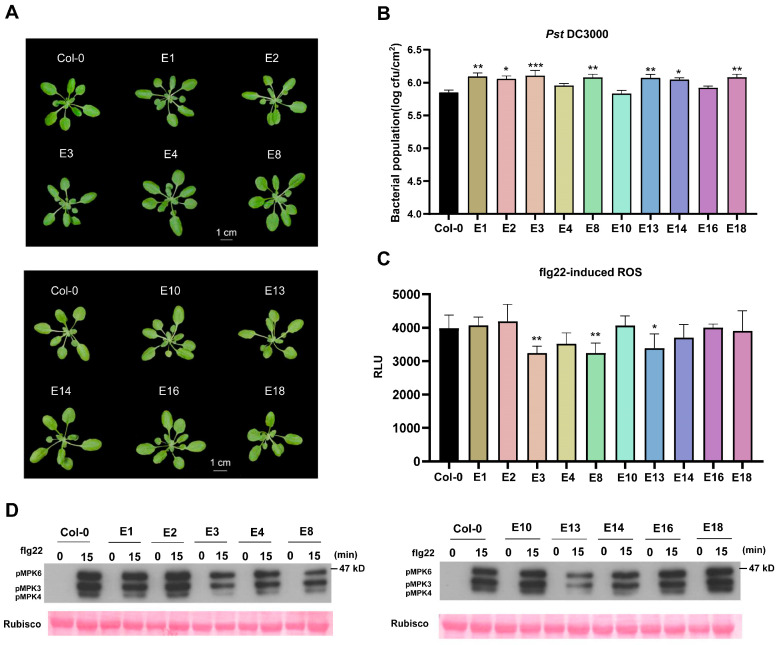
Analyses of the immune and disease resistance phenotype in Arabidopsis transgenic plants expressing the candidate virulence effectors. (**A**) Morphology of the stable transgenic Arabidopsis plants expressing the candidate virulence effectors. The indicated protein was introduced into Arabidopsis plant by *Agrobacterium*-mediated transformation. Photographs were taken of 4-week-old stable transgenic plants at the T3 generation. EV, empty vector. Bar, 1 cm. (**B**) Evaluation of *Pst* DC3000 resistance in transgenic Arabidopsis plants expressing the candidate effectors. Leaves of the indicated transgenic plants were infected with *Pst* DC3000 and the bacterial population was determined 3 days later (Mean ± SD, *n* ≥ 6, one-way ANOVA; * *p* < 0.05, ** *p* < 0.01, *** *p* < 0.001). (**C**) Examination of flg22-induced ROS burst in the transgenic Arabidopsis expressing the effectors. ROS burst induced by flg22 (1 μM) was examined in the indicated transgenic plants and peak RLU values were recorded (Mean ± SD, *n* ≥ 6, one-way ANOVA; * *p* < 0.05, ** *p* < 0.01). (**D**) Analyses of flg22-induced MAPK activation in the transgenic Arabidopsis expressing the effectors. Seedlings of the indicated genotypes were sprayed with flg22 (1 μM) for 0 and 15 min, and MAPK activation was examined by anti-pERK immunoblotting. Rubisco was used as an internal control.

To eliminate potential artifacts from the transient expression system and verify the biological relevance of our findings, we generated stable Arabidopsis transgenic lines expressing these ten candidate effectors and obtained homozygous T3 generation plants (Figure 6A). None of the transgenic lines showed visible developmental defects, confirming that these effectors do not intrinsically disrupt plant growth (Figure 6A). qPCR analysis confirmed that the transcripts of all these candidate effectors were successfully detected in the transgenic lines (Figure S7). To validate the effect of candidate virulence effectors on plant disease resistance and immunity, we examined the resistance of transgenic plants to *Pst* DC3000 and PTI activation. Pathogen infection assays demonstrated that multiple effector-expressing lines exhibited significantly enhanced susceptibility to *Pst* DC3000, with E1, E2, E3, E8, E13, E14, and E18 showing particularly strong immunosuppressive activity (Figure 6B).

We further examined the flg22-induced ROS burst in these transgenic lines and found that E3, E8, and E13 significantly inhibited the flg22-induced ROS burst (Figure 6C). The MAPK pathway is one of the most conserved signaling cascades in eukaryotes. Upon perception of PAMPs, plants rapidly activate the MAPK cascade [37]. We assessed flg22-induced MAPK phosphorylation in the transgenic lines using an anti-pERK antibody. E3, E8, E13, and E14 substantially suppressed the flg22-triggered MAPK activation (Figure 6D).

### 3.6. Effector E3 May Suppress ROS Accumulation by Targeting NADPH Oxidases

**Figure 7:**
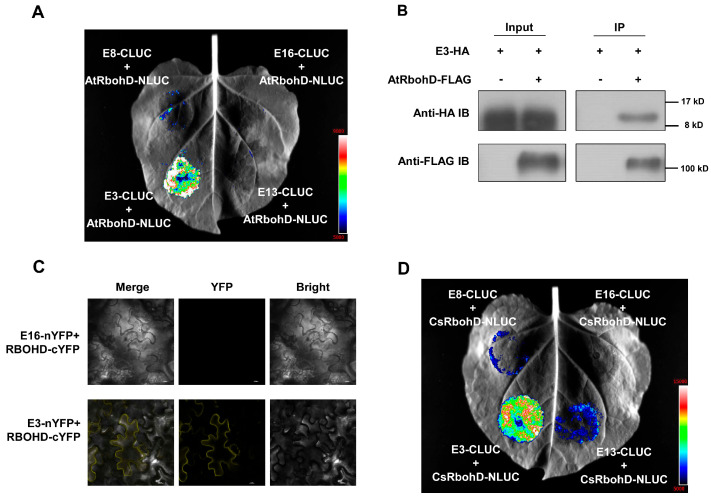
E3 interacts with plant NADPH oxidases. (**A**) E3, but not E8 or E13, interacts with AtRbohD in *N. benthamiana*. The indicated Nluc and Cluc vectors were co-expressed in *N. benthamiana* plants using *Agrobacterium*-mediated transient expression assay and subjected to luciferase complementation imaging assays. E16 was used as a negative control. (**B**) E3 interacts with AtRbohD as determined by coimmunoprecipitation (co-IP) assays. The indicated proteins were transiently expressed in *N. benthamiana* using an *Agrobacterium*-mediated expression assays, and protein interactions were examined using co-IP assays. (**C**) E3 interacts with AtRbohD in a bimolecular fluorescence complementation (BIFC) assay. The indicated proteins were transiently expressed in *N. benthamiana* using an *Agrobacterium*-mediated expression assay, and protein interactions were examined using BIFC assays. (**D**) E3 interacts with citrus NADPH oxidase CsRbohD (Cs8g12000). The indicated Nluc and Cluc vectors were coexpressed in *N. benthamiana* plants and subjected to LCI assays.

Finally, we conducted preliminary investigations to identify potential targets of these effectors. The perception of PAMPs by PRRs activates downstream immune responses by interacting with certain key downstream regulators, such as the NADPH oxidase RbohD, which is responsible for the direct generation of ROS [6]. We found that E3, E8, and E13 strongly suppress PAMP-induced ROS production. Therefore, we used a luciferase complementation imaging assay to test for potential protein interactions between effectors and AtRbohD. The results showed a strong interaction between E3 and AtRbohD, but no obvious interactions were detected between E13 or E8 and AtRbohD (Figure 7A). A co-immunoprecipitation assay further confirmed that E3 interacted with AtRbohD (Figure 7B). The BIFC assay demonstrated that E3 and AtRbohD may interact at the cell membrane (Figure 7C). We finally determined whether E3 interacts with NADPH oxidase in citrus plants. To verify this, we examined the interaction between E3 and CsRbohD (Cs8g12000), an NADPH oxidase in citrus [38]. E3 strongly interacted with citrus CsRbohD proteins (Figure 7D). Based on these findings, we propose that E3 likely targets NADPH oxidases to inhibit ROS generation, thereby suppressing the ROS-mediated immune response.

## 4. Discussion

In this study, we used SignalP 6.0 to predict secretion signals in CLas effector proteins, identifying 40 candidate effectors. Using a combination of transient expression in *N. benthamiana* and stable transgenic expression in Arabidopsis, we evaluated the effects of these candidate effectors on plant PTI. The assessed immune responses included INF1-induced cell death, flg22- and chitin-triggered ROS bursts, and resistance to the bacterial pathogen *Pst* DC3000. We identified ten CLas virulence effectors capable of significantly suppressing plant immunity. Among these, effector E3 was found to interact with the key ROS-producing enzyme NADPH oxidase, thereby inhibiting the ROS burst. This is a finding that provides initial insights into its mechanistic role.

Functional characterization of the ten candidate effectors revealed a sophisticated strategy used by CLas to disarm host immunity. These effectors exhibit both specialized and overlapping functions, suggesting that they target common and specific nodes of the plant immune network. The suppression of multiple PTI responses by E3, E13, and E14 implies that they potentially interfere with upstream convergent signaling components, such as PRRs, co-PRRs, and RLCKs [6]. Conversely, the more specific inhibition of either cell death (e.g., E10) or ROS burst (e.g., E1, E8, E16, E18) by other effectors indicates the targeting of components specific to certain immune signaling components, such as RbohD. This functional redundancy and diversification are characteristic of successful pathogens, enabling them to disrupt multiple layers of defense simultaneously and robustly. This is crucial for obligate parasites, such as CLas, that depend entirely on the host for survival. Consistent upregulation of these effectors during infection further underscores their importance in establishing host compatibility.

The validation of immunosuppressive phenotypes in stable Arabidopsis transgenic lines reinforces the biological relevance of these effectors as virulence factors, mitigating concerns regarding artifacts from transient expression systems. The correlation between PTI suppression and enhanced susceptibility to *Pst* DC3000 in transgenic plants strongly supports the conclusion that these effectors compromise basal immunity. However, it is important to acknowledge the limitations of these heterologous pathosystems. Although *N. benthamiana* and Arabidopsis are powerful tools for screening, the natural interaction between CLas and citrus may involve host-specific factors and immune components that are not fully recapitulated here. Therefore, the primary function of these effectors in suppressing core PTI has been established. However, their precise roles and relative contributions during CLas infection in citrus remain to be investigated. Nonetheless, this study provides critical candidate virulence effectors and functional clues for the future exploration of CLas pathogenesis.

Among the ten candidate effectors identified in this study, E4 (SDE2), E8 (SDE4405), E13 (SDE1), E14 (SDE19), and E16 (CLas0185) had been previously reported to suppress host immune responses [20,39,40,41,42,43,44]. E13 (SDE1) targets citrus papain-like cysteine proteases (PLCPs) [20], whereas E14 (SDE19) targets the endoplasmic reticulum GEF protein Sec12, disrupting vesicle trafficking, inhibiting the secretion of defense-related proteins, and promoting pathogen infection [44]. The successful replication of known effector functions in our experimental system strongly validates the reliability and effectiveness of our screening strategy. More importantly, we identified five previously unreported virulence effectors (E1, E2, E3, E10, and E18). Among these, E3 is one of the most potent suppressors of the ROS burst. We demonstrate that E3 interacts with plant NADPH oxidases. However, the precise molecular mechanisms underlying ROS inhibition by E3 require further investigation. E3 possesses definite secretory capabilities [18]. According to Ma et al. (2022), Huanglongbing symptoms may be associated with ROS accumulation leading to autoimmune-like defects [45]. We propose that E3 may facilitate CLas infection by targeting NADPH oxidase, thereby blocking ROS-mediated immune responses and creating a favorable environment for pathogen colonization within the host.

## 5. Conclusions

In this study, we used SignalP 6.0 for effector secretion prediction and used *N. benthamiana* and Arabidopsis expression systems to analyze the impact of candidate effectors on microbial pattern-induced immune responses and disease resistance. We identified ten CLas-secreted effectors as potential key virulence factors that undermine plant basal immunity by suppressing core PTI responses. E3 interacts with plant NADPH oxidases, inhibiting ROS production and disrupting ROS-mediated immune responses. These findings substantially advance our understanding of CLas pathogenesis and provide valuable guidance for the development of CLas-resistant plants, particularly citrus species, using technologies such as host-induced gene silencing. Future research should focus on identifying the host targets of these virulence effectors and elucidating their roles in citrus resistance against CLas, as these targets may have potential applications in improving plant resistance through genetic modification approaches.

## Figures and Tables

**Table 1:** Strains and plasmids used in the study.

Plasmids/Strains	Source
pCAMBIA1300-35S-HA-RBS	[24]
pCAMBIA1300-35S-FLAG-RBS	[24]
pCAMBIA1300-35S-HA-Nluc-RBS	[25]
pCAMBIA1300-35S-Cluc-RBS	[25]
pEarleygate201-35S-nYFP-RBS	[26]
pEarleygate201-35S-cYFP-RBS	[26]
GV3101	TransGenBiotech, Beijing, China
DH5α	TransGenBiotech, Beijing, China
*Pst* DC3000	[27]
*Pst* DC3000 *ΔhopQ1*	[28]
*Pst* DC3000 *hrcC*	[29]

## Data Availability

The original contributions presented in this study are included in the article/Supplementary Material. Further inquiries can be directed to the corresponding authors.

## Supplementary Materials

The following supporting information can be downloaded at: https://www.mdpi.com/article/10.3390/plants15020308/s1, Figure S1. Examination of the protein expression levels of the effector proteins in *N. benthamiana*; Figure S2. Three effectors caused the cell death phenotype in *N. benthamiana* plants; Figure S3. Examination of the effect of effectors on INF1-induced cell death phenotype in *N. benthamiana*; Figure S4. Analyses of the effect of transient expression of the effectors on ROS burst induced by flg22 in *N. benthamiana*; Figure S5. Analyses of the effect of transient expression of the effectors on ROS burst induced by chitin in *N. benthamiana*; Figure S6. Analyses of the effect of transient expression of the effectors on *N. benthamiana* resistance to *Pseudomonas syringae* pv. *tomato* DC3000 (*Pst* DC3000) *ΔhopQ1*; Figure S7. Construction of transgenic Arabidopsis plants expressing 10 virulence effectors; Table S1. Primers used for this study, Table S2. Prediction of signal peptide of candidate effectors by SignalP 6.0, Table S3. A list of 40 candidate secreted effectors of *Candidatus* Liberibacter asiaticus (CLas).
