# Supplementary material for: Functional Analysis Identifies Multiple Effectors of Candidatus Liberibacter Asiaticus Suppressing Plant Pattern-Triggered Immunity
Source: Plants (Basel). 2026 Jan 20;15(2):308. doi: 10.3390/plants15020308 (PMC12845134; doi:10.3390/plants15020308)
Supplement: Supplementary file 1 [file plants-15-00308-s001.zip › plants-3997297-supplementary.pdf]

plants

MDPI

Supplementary Data

Functional Analysis Identifies Multiple Effectors of *Candidatus* Liberibacter Asiaticus Suppressing Plant Pattern-Triggered Immunity

Zhuoyuan He [1,†], Hongyan Li [1,†], Zonghui Zhao [1], Desen Wang [2], Hong Wu [1], Mei Bai [1], Xiangxiu Liang [1,*] and Jian-Bin Yu [1,*]

* Correspondence: liangxiangxiu@scau.edu.cn (X.L.); ybb1129@126.com (J.-B.Y.)

Keywords: *Candidatus Liberobacter asiaticus*; effectors; virulence; plant immunity; disease resistance

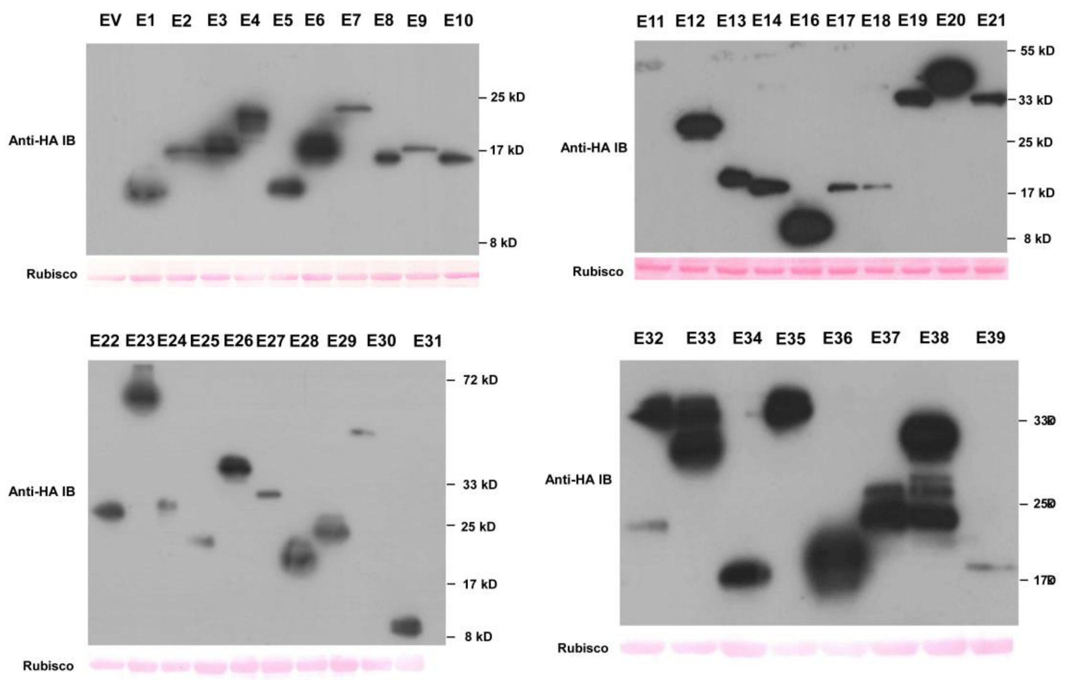

Figure S1. Examination of the protein expression levels of the effector proteins in *N. benthamiana*. The indicated constructs were expressed in *N. benthamiana* by *Agrobacterium*-mediated transient expression and protein expression levels were examined by anti-HA immunoblotting.

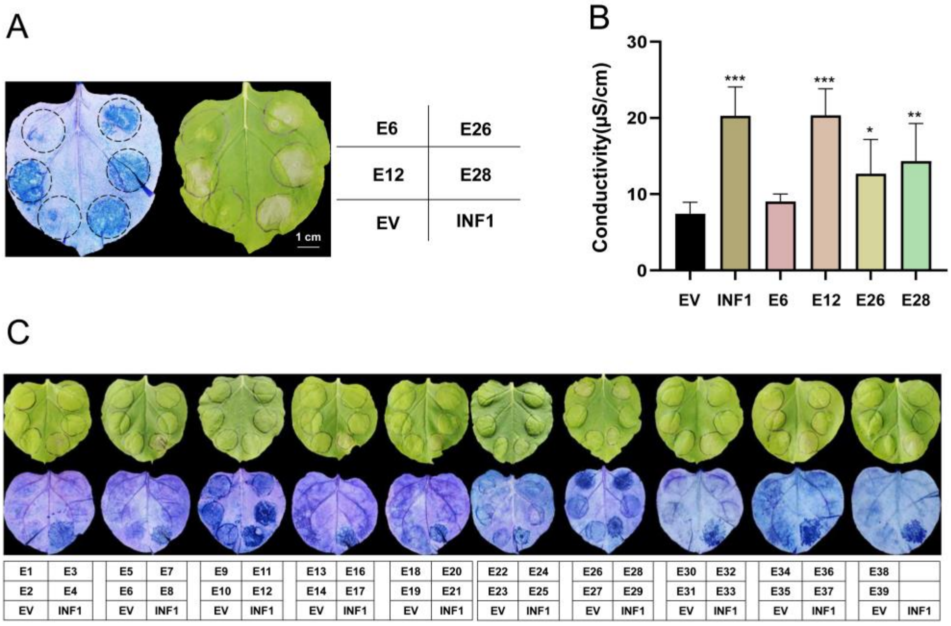

Figure S2. Three effectors caused the cell death phenotype in *N. benthamiana* plants (A-B) Expression of E12, E26 and E28 in *N. benthamiana* plants triggered cell death phenotype. The indicated effectors were expressed in *N. benthamiana* by *Agrobacterium*-mediated transient expression for 3 days. The cell death phenotype was visualized by trypan blue staing (A) and examined by ion leakage assay (B) (Mean±SD, n≥6, one-way ANOVA; **p*< 0.05, ***p*< 0.01, ****p*<0.001). INF and E6 was used as a positive and negative control, respectively. EV, empty vector. (C) Examination of cell death phenotypes induced by all the cloned candidate effectors in *N. benthamiana*. A number of 38 cloned effectors were expressed in *N. benthamiana* by *Agrobacterium*-mediated transient expression for 3 days. The cell death phenotype was visualized by trypan blue staining.

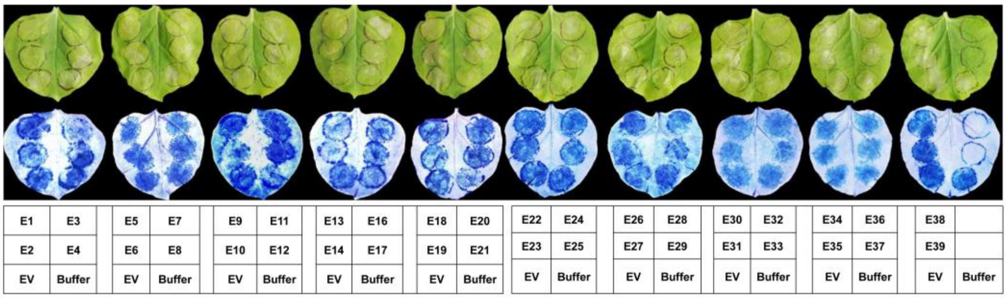

Figure S3. Examination of the effect of effectors on INF1-induced cell death phenotype in *N. benthamiana*. Analyses of the effect of transient expression of all the cloned candidate effectors on INF-induced cell death in *N. benthamiana* plants. The indicated effectors were transiently expressed in *N. benthamiana* for 1 day, and infiltrated with *Agrobacterium* carrying INF1. The cell death phenotypes were visualized by trypan blue staining 3 days later.

https://doi.org/10.3390/plants15020308

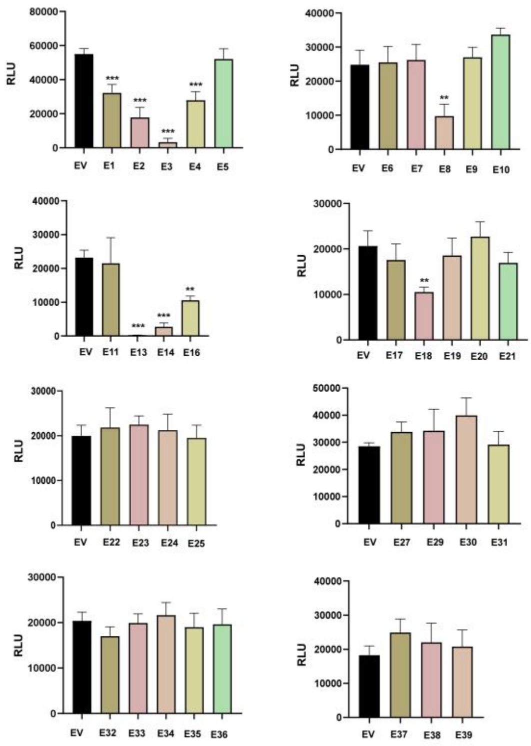

Figure S4. Analyses of the effect of transient expression of the effectors on ROS burst induced by flg22 in *N. benthamiana*. The indicated effector was expressed in *N. benthamiana* plants by *Agrobacterium*-mediated transient expression for 2 days. ROS production induced by flg22 (1 μM) was examined and peak relative luminescence unit (RLU) values are recorded (Mean±SD, n≥6, one-way ANOVA; $^{**}p < 0.01$, $^{***}p < 0.001$). **.**

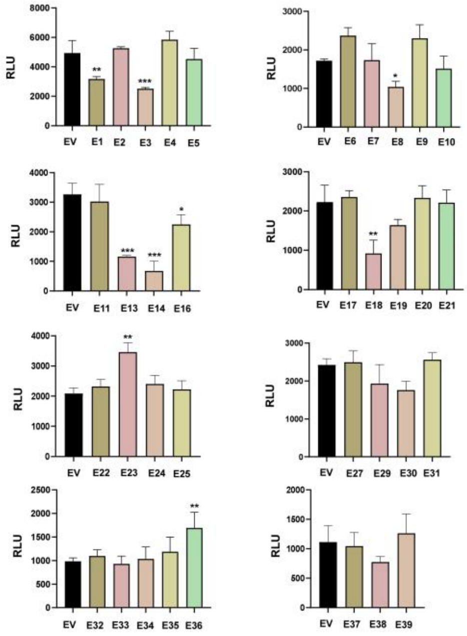

Figure S5. Analyses of the effect of transient expression of the effectors on ROS burst induced by chitin in *N. benthamiana*. The indicated effector was expressed in *N. benthamiana* plants by *Agrobacterium*-mediated transient expression for 2 days. ROS production induced by chitin (200 μg/mL)

was examined and peak relative luminescence unit (RLU) values are recorded (Mean±SD, n≥6, one-way ANOVA; **p*< 0.05, ***p*< 0.01, ****p*< 0.001). .

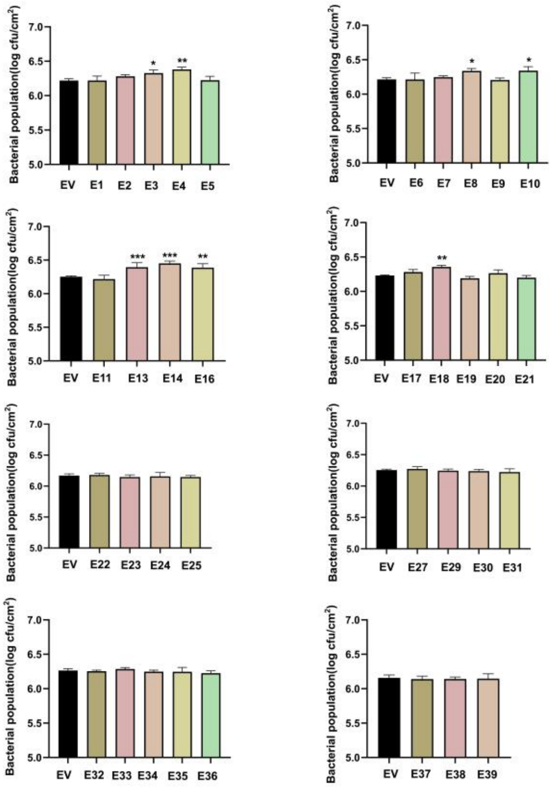

Figure S6. Analyses of the effect of transient expression of the effectors on *N. benthamiana* resistance to *Pseudomonas syringae* pv. *tomato* DC3000 (*Pst* DC3000) *Δhop Q1*. The indicated effector was expressed in *N. benthamiana* plants by *Agrobacterium*-mediated transient expression assay for 2 days. The leaves were infiltrated with *Pst* DC3000 *ΔhopQ1*, and the bacterial number was determined 3 days after inoculation (Mean±SD, n≥6, one-way ANOVA; **p*< 0.05, ***p*< 0.01, ****p*< 0.001).

https://doi.org/10.3390/plants15020308

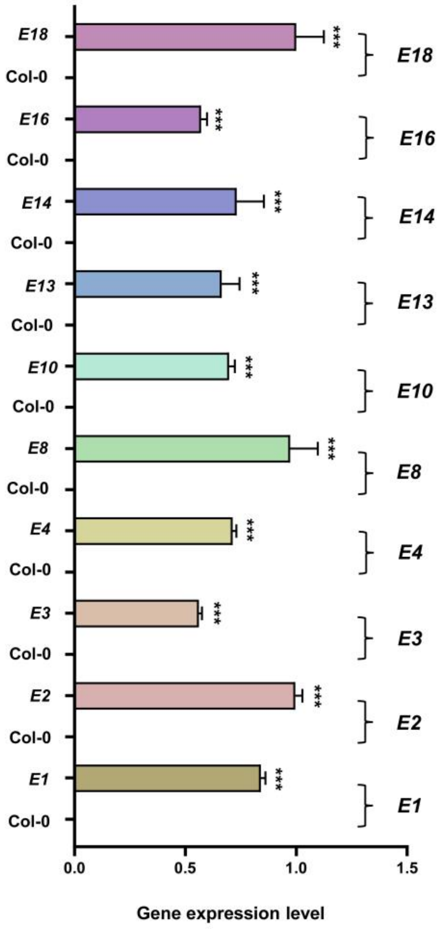

Figure S7. Construction of transgenic Arabidopsis plants expressing 10 virulence effectors. The transcription levels of the effectors in transgenic Arabidopsis were examined by qPCR analysis. Total RNA of the indicated plants was extracted by subjected to qPCR analysis by using specific primers (Mean±SD, student t test, ***p< 0.001). *ACT2* was used as an internal control.

Table S1. Primers used for this study.

Primers	Sequence 5′ to 3′
E1-F	CGGGGGACGAGCTCGGTACCATGTATACCAAAAGTTTGTTAATGGTAGCT
E1-R	ACATCGTATGGGTAGTCGACTTGGTAGCGCCTTTCTCTTG
E2-F	CGGGGGACGAGCTCGGTACCATGAGAGCTAAAACATTACTGGC
E2-R	ACATCGTATGGGTAGTCGACGTAATCCGATACTTTCTCCCC
E3-F	CGGGGGACGAGCTCGGTACCATGAAAATGAATATTATAAAAAAAATTTTA
E3-R	ACATCGTATGGGTAGTCGACCGAGCTTTTATTTGGCTTAAC
E4-F	CGGGGGACGAGCTCGGTACCATGAACTTCAGAATAGCGATG
E4-R	ACATCGTATGGGTAGTCGACGGACATAAACCCTTTGACG
E5-F	CGGGGGACGAGCTCGGTACCATGAATGCCAAGGGATTAATAG
E5-R	ACATCGTATGGGTAGTCGACTTGTTCCTTTATTGCTATTTCTATCTTTC
E6-F	CGGGGGACGAGCTCGGTACCATGACAATATCAAAAAATCAAGCCATTC
E6-R	ACATCGTATGGGTAGTCGACTCTTTCCCATTCTCTAACGT
E7-F	CGGGGGACGAGCTCGGTACCATGGTACGTGTTTTTTGTGC
E7-R	ACATCGTATGGGTAGTCGACGAATTTATAAACATAATCAAGACCTATC
E8-F	CGGGGGACGAGCTCGGTACCATGAAAGCTAAAATATTGATGACATCTGC
E8-R	ACATCGTATGGGTAGTCGACATTTGATGGGGGCACAGTGA
E9-F	CGGGGGACGAGCTCGGTACCATGAAAAAGTATTTCACAATTTTAACAATG
E9-R	ACATCGTATGGGTAGTCGACGTAGCGGTGTCCGTTGTTTT

E10-F	CGGGGGACGAGCTCGGTACCATGACTATTAAGAAAGTACTAATTGCTTCA
E10-R	ACATCGTATGGGTAGTCGACATCACTAGATAGTTTCGCACCCTTTGTAAG
E11-F	CGGGGGACGAGCTCGGTACCATGGATATCAAGGGATTAATAGTGGCTTC
E11-R	ACATCGTATGGGTAGTCGACTTTTCTTAAAAAATTTTGTGATCTAAGTTC
E12-F	CGGGGGACGAGCTCGGTACCATGAAATCAAAAAATATTCTCATTGTATCAACG
E12-R	ACATCGTATGGGTAGTCGACAAATGCTACGTCCCATAGCT
E13-F	CGGGGGACGAGCTCGGTACCATGGGCAGTAGTTTTGGTTGTTGT
E13-R	ACATCGTATGGGTAGTCGACAGACTGCTCCAACATTTTTCTATGG
E14-F	CGGGGGACGAGCTCGGTACCATGAGTAAGTTTGTGGTGAGG
E14-R	ACATCGTATGGGTAGTCGACATTGTTTAAGCCTCCAAAGAGC
E15-F	CGGGGGACGAGCTCGGTACCATGCAAGCTCGGTGCTTTTTATCCTTATCG
E15-R	ACATCGTATGGGTAGTCGACTTTATAAAATATGATTGACGTAGAAGGGGT
E16-F	CGGGGGACGAGCTCGGTACCATGGATCATAGAAAGAAAACGATCG
E16-R	ACATCGTATGGGTAGTCGACAATCACCAAGGTCACGTCGA
E17-F	CGGGGGACGAGCTCGGTACCATGAAAAAAACACAATTACTTTTGCC
E17-R	ACATCGTATGGGTAGTCGACCTCTTTCTCATTTTGCTCAATGG
E18-F	CGGGGGACGAGCTCGGTACCATGCGCTTTAAAACAAAACA
E18-R	ACATCGTATGGGTAGTCGACTTGTTCAAGGGTTGTGGTTT
E19-F	CGGGGGACGAGCTCGGTACCATGTATAAAATATTAGCTGTATGC
E19-R	ACATCGTATGGGTAGTCGACATTATTCATAAATCTCTCTAGTTGACG
E20-F	CGGGGGACGAGCTCGGTACCATGATTTTTCGTATAGCTTTTTTG
E20-R	ACATCGTATGGGTAGTCGACTTGAATTATAATCTCTGATTGAAGGGCGCC
E21-F	CGGGGGACGAGCTCGGTACCATGTTACGTTATTTTATATGTTTGTTGTTC
E21-R	ACATCGTATGGGTAGTCGACAAATAAAGTATCCACAATTTTCG
E22-F	CGGGGGACGAGCTCGGTACCTTGAAGTACCGAGTTTTATTATTG
E22-R	ACATCGTATGGGTAGTCGACTTTGCTGATCTGAACGTCAT
E23-F	CGGGGGACGAGCTCGGTACCATGATAGCACTGTGTTTAATAGCGTCG
E23-R	ACATCGTATGGGTAGTCGACCTGAACACTCTTAGTGTCTTC
E24-F	CGGGGGACGAGCTCGGTACCTTGCCGTTAAGTGGTTATAGCG
E24-R	ACATCGTATGGGTAGTCGACTTCTTCCATTTTCGGTGGATCC
E25-F	CGGGGGACGAGCTCGGTACCATGTGTAGAAAAATTATCTTTGCACTTAC
E25-R	ACATCGTATGGGTAGTCGACATTATTTATAAATGGGCAGAGCAGATCGCC
E26-F	CGGGGGACGAGCTCGGTACCGTGAGATTTTTTTTTGTAGTTC
E26-R	ACATCGTATGGGTAGTCGACACCGCTCTTTCCAACTGCAA
E27-F	CGGGGGACGAGCTCGGTACCATGAGTGCTTTTTTGAATAGTATAC
E27-R	ACATCGTATGGGTAGTCGACTGGACGCCCGTCATAACGTA
E28-F	CGGGGGACGAGCTCGGTACCATGAAATTACTTTTTTCCAAAATTGCTCC
E28-R	ACATCGTATGGGTAGTCGACGGCTCCTTCTTTTTTATCCTC
E29-F	CGGGGGACGAGCTCGGTACCATGAAAAGATTGAAATATCAAATTA
E29-R	ACATCGTATGGGTAGTCGACATCCTCTTTTGTGTTTTCTGCTATC
E30-F	CGGGGGACGAGCTCGGTACCATGAATATTCTACGAGCCATTTTTTCTC
E30-R	ACATCGTATGGGTAGTCGACCGGATTATATCCACTTCCAA
E31-F	CGGGGGACGAGCTCGGTACCATGAATACAAGAATAATAGGAACCG
E31-R	ACATCGTATGGGTAGTCGACTCTTCTGGATTGATTTTTACC
E32-F	CGGGGGACGAGCTCGGTACCATGAGTAATAAGTCTTGTATTAGTTT
E32-R	ACATCGTATGGGTAGTCGACATATTCTTTTCTATAATTTTTCTGTCTTTC
E33-F	CGGGGGACGAGCTCGGTACCATGAAATATAAGATCGCGATTATC
E33-R	ACATCGTATGGGTAGTCGACTATAAAGAAGTGCATAGGATATTTTGC
E34-F	CGGGGGACGAGCTCGGTACCATGAAGAAGTATATCACATTATTAACAG
E34-R	ACATCGTATGGGTAGTCGACCTGGGATCGGTAGTTTCGAT
E35-F	CGGGGGACGAGCTCGGTACCATGATAAGGAAATATGTTTTAGCTC
E35-R	ACATCGTATGGGTAGTCGACAAAATCATAATCGATGCTGATAGGAC
E36-F	CGGGGGACGAGCTCGGTACCATGTTTTGGATTGCAAAAAAATTTTTTTGG

https://doi.org/10.3390/plants15020308

E36-R	ACATCGTATGGGTAGTCGACATGATGCGACGGCAAAGGAG
E37-F	CGGGGGACGAGCTCGGTACCATGCATTTTTATCGTTTTATTCTC
E37-R	ACATCGTATGGGTAGTCGACCCGAGAAATTATAACTTTATCAC
E38-F	CGGGGGACGAGCTCGGTACCATGAGAGATATAAGAAAAATTAG
E38-R	ACATCGTATGGGTAGTCGACAAAGCGTAAAACCACACCAG
E39-F	CGGGGGACGAGCTCGGTACCATGCATTTTAAAATAAAACGATTTCTC
E39-R	ACATCGTATGGGTAGTCGACAGAGAGGTCTTCCTTCAGTT
E40-F	CGGGGGACGAGCTCGGTACCATGGTTAGTAAAAACATAGG
E40-R	ACATCGTATGGGTAGTCGACTCTTTGGATTAGTTTATTAAAG
E1-qF	TGTTATCTTCGGTTGCAATA
E1-qR	CTTCTTTAGACGGGCCTTT
E2-qF	CTCTTGTGACGACCGCTATT
E2-qR	CGCGTTGATTAGCTTGTTCC
E3-qF	GGTGCAACGGCTATTGAGTA
E3-qR	TCCGTTGAGATCGTCTGGTA
E4-qF	GCTATGTGTTGAGTGTCCGT
E4-qR	TATTCTCGCATTCGGCACAA
E8-qF	TAGCACAGGAACGTGTTGAG
E8-qR	AAGCGTTCGCCAATTCATCT
E10-qF	TGCTTCAACTTTATTATCCCTCTGT
E10-qR	AAGCCTACAAACGGCATCAC
E13-qF	ATACTCCGCGTGTTCCTGAT
E13-qR	CGAGTGCGACTAATGTGCTT
E14-qF	TAGCTGCCAATGAGCACTCT
E14-qR	AAGCACCTCTCGTGTATCGT
E16-qF	TCCACATTAGCTGGCTGTGA
E16-qR	ATCACCAAGGTCACGTCGAT
E18-qF	ACCTTATTAGGTAGCTGTGC
E18-qR	TGTAATTTATCCATCCTGTGC
gryA-qF	CAATGTGCTGGTCAATGGTG
gryA-qR	AATCTCCATCAAGGCATCCAG
ACT2-qF	TGCTGGATTCTGGTGATGGT
ACT2-qR	AGTAAGGTCACGTCCAGCAA

Table S2. Prediction of signal peptide of candidate effectors by SignalP 6.0.

Table S3. A list of 40 candidate secreted effectors of *Candidatus* Liberibacter asiaticus (CLas)**.** The signal peptide was predicated with SignalP 6.0 online software. The secretion probability threshold was set as >20%. Effectors with putative signal peptide domain was considered as candidate virulence effectors.

Number	Gene	Amino acids	Molecular weight (kDa)	Probability predicated by SignalP 6.0
E1	CLIBASIA_00470	51	5.85	0.368482
E2	CLIBASIA_02215	120	13.8	1
E3	CLIBASIA_03085	120	12.76	0.68145
E4	CLIBASIA_03230	162	17.77	0.999527
E5	CLIBASIA_03915	41	4.51	0.999007
E6	CLIBASIA_04025	96	11.04	1
E7	CLIBASIA_04320	215	24.63	0.999776
E8	CLIBASIA_04405	121	13.54	1
E9	CLIBASIA_04410	122	14.23	0.758037
E10	CLIBASIA_05640	68	7.56	0.999688
E11	CLIBASIA_04530	85	9.13	1

E12	CLIBASIA_04560	195	21.77	1
E13	CLIBASIA_05315	154	17	0.959184
E14	CLIBASIA_05320	85	9.48	0.999518
E15	CLIBASIA_00420	160	17.77	1
E16	CLIBASIA_00185	68	7.7	1
E17	CLIBASIA_00525	97	11.46	1
E18	CLIBASIA_00530	97	11.28	1
E19	CLIBASIA_01135	309	35.04	0.999766
E20	CLIBASIA_01305	369	39.17	0.999734
E21	CLIBASIA_02120	294	33.07	0.999421
E22	CLIBASIA_02145	210	23.5	0.999535
E23	CLIBASIA_02160	647	70.5	0.99945
E24	CLIBASIA_02305	200	22.67	0.982182
E25	CLIBASIA_02470	131	14.77	0.999676
E26	CLIBASIA_02970	345	38.73	0.999679
E27	CLIBASIA_03160	181	20.54	0.999735
E28	CLIBASIA_04030	88	9.26	1
E29	CLIBASIA_04040	159	17.19	0.999912
E30	CLIBASIA_04145	452	51.2	1
E31	CLIBASIA_04250	49	5.28	0.999985
E32	CLIBASIA_04290	302	35.8	0.260945
E33	CLIBASIA_04330	229	26.03	0.200232
E34	CLIBASIA_04425	125	14.88	0.99954
E35	CLIBASIA_04520	304	35.34	0.998474
E36	CLIBASIA_04580	116	13.24	0.999611
E37	CLIBASIA_04735	162	18.57	0.993296
E38	CLIBASIA_05150	225	24.82	0.998979
E39	CLIBASIA_00520	117	13.52	1
E40	CLIBASIA_05460	42	4.7	0.999948

https://doi.org/10.3390/plants15020308
